# Editorial: Nutritional impacts the health and physiology of the avian gastro-intestinal tract

**DOI:** 10.3389/fphys.2024.1470024

**Published:** 2024-08-21

**Authors:** Krystyna Pierzchała-Koziec, Oluyinka Olukosi, Ramesh Selvaraj, Revithi Shanmugasundaram, Elham Assadi Soumeh, Colin G. Scanes

**Affiliations:** ^1^ Department of Animal Physiology and Endocrinology, University of Agriculture in Krakow, Krakow, Poland; ^2^ Department of Poultry Science, University of Georgia, Athens, GA, United States; ^3^ Toxicology and Mycotoxin Research Unit, U.S. National Poultry Research Center, Agricultural Research Service (USDA), Athens, GA, United States; ^4^ School of Agriculture and Food Sustainability, Faculty of Science, University of Queensland, Gatton, QLD, Australia; ^5^ Department of Biological Sciences, University of Wisconsin, Milwaukee, WI, United States

**Keywords:** nutrition, gastrointestinal tract, microbiome, birds, health, botanicals 1, nutritional

## 1 Introduction

There is a considerable body of information on the nutritional requirements of poultry and other avian species. Moreover, the avian gastro-intestinal tract marked similarities to that of mammals. Kuzmina et al. contribute an Opinion article on activities of intestinal enzymes. Unique features of the physiology of the avian gastro-intestinal tract include the separate glandular and muscular regions of the stomach, names the proventriculus and gizzard, the presence of a crop, a feed storage organ extending from the esophagus and the presence of two ceca. In addition, there are developmental differences between birds and viviparous mammals with bird embryos growing in large yolky eggs. In the present topic, Kuzmina discusses the importance of the embryonic yolk sac in birds in comparison with other vertebrates.

Research on avian nutritional physiology and health is increasingly employing anti-oxidative and molecular parameters together with traditionally employed parameters including body weight, feed efficiency and organ weights.

Among the objectives of this topic are the following:

1. To address both unique aspects of avian nutritional physiology and some poultry specific management techniques.
2. To determine effects of pathogens on the functioning of avian gastro-intestinal tract and how adverse effects be mitigated.
3. To understand the nutritional impacts on gut microbiota and the cross-talks between the microbes and the host.
4. To develop nutritional and other strategies (botanics, probiotics etc.) to reduce food borne colonization in poultry and reduce prevalence of food borne pathogens in poultry products.

## 2 Discussion

The importance of dietary arginine is reviewed (Fathima et al.). Not only is arginine required for protein synthesis but also is necessary to production of nitric oxide (NO), functioning of the immune functioning, gut microbiota and regulating mammalian target of rapamycin (mTORC) (Fathima et al.).

There were no effects of either dietary calcium and/or lipopolysaccharide (LPS) challenges on serum concentrations of ionized calcium and inorganic phosphate in hens towards the end of their production cycle (Li et al.). However, serum concentrations of alkaline phosphatase (ALP) were markedly elevated in hens receiving a low calcium feed but depressed those in hens challenged with LPS (Li et al.). Moreover, bone concentrations of calcium were increased in hens receiving LPS challenges (Li et al.). Splenic expression of inflammatory cytokines, TNF-α, interleukin (IL)-1β and IL-6, IL-10, IL-17 and interferon γ, were increased in hens receiving LPS challenges but unaffected by calcium level in the feed (Li et al.). Moreover, tibial expression of TNF-α, IL-1β, IL-6, IL-10, IL-17 and interferon γ were increased in hens receiving LPS challenges but, with the exception of TNF-α, unaffected by calcium level in the feed (Li et al.). Tibial expression of osteoblast metabolism-related genes: *ALP* and *Ocn* was influenced by calcium level or injection of LPS. Unexpectedly, mRNA gene expression of *FGF23* was not changed by the treatments (Li et al.). Expression of ALP was increased in hens receiving a low calcium feed but decreased following challenge with LPS (Li et al.). Expression of osteocalcin was increased in hens on a low calcium feed or following LPS challenge. The effect of LPS were greater in hens on a low calcium diet (Li et al.). The increase in tibial expression of TNF-α was greater in hens on a low compared to recommended levels of calcium (Li et al.).

Increasing numbers of poultry are raised without antibiotics. There is growing attention to using dietary supplements to replace antibiotics, to alter intestinal microbial populations, to stimulate antioxidative and immune systems and to improve overall intestinal health. Plant based and other dietary microingredients have been increasing considered as dietary supplements for humans, livestock, companion animals and poultry. These botanics can have beneficial effects including “*anti-inflammatory, anti-oxidative and pro-gut health*” (reviewed: Sulaiman et al.).

Feeding hens and/or pullets a flaxseed enriched diet increased the yolk concentrations of the following long-chain omega-3 polyunsaturated fatty acids (n-3 FAs): alpha-linolenic acid (C18:3 n-3), eicosapentaenoic acid (C20:5 n-3), and docosapentaenoic acid (C22:5 n-3) (Whittle et al.). Moreover, there was an increase in brain weight but a decrease in brain concentrations of alpha-linolenic acid (C18:3 n-3) (Whittle et al.).

Both intraperitoneal and intracerebroventricular administration of the polyphenolic phytocompound, oleuropein, reduce feed intake in 4-day-old chickens (Sulaiman et al.). Moreover, plasma concentrations of glucose were depressed following administration of oleuropein (Sulaiman et al.).

Addition of a mixture of wheat germ, hops and grape seed extract was followed by increased growth rate in broiler chickens and improved feed efficiencies (Zou et al.). Based on fecal microorganisms, there were shifts in the gastro-intestinal microbiomes with decreases in numbers of both *Salmonella* and *E. coli* but increases in those of *Lactobacillus* (You et al., 2023).

What is unique in this study was the measurement of release of ammonia and hydrogen sulfide from manure; these being environmental pollutants released by poultry and livestock production (You et al., 2023).

Addition of pretreated Chinese herbal medicine to feed was followed by increased rates of egg laying and improved feed efficiency in laying hens towards the end of their production cycle (Zou et al.). Accompanying this were shifts in antioxidative systems including both enzyme activity and gene expression in plasma, liver, magna of oviduct and uterus (a region of the oviduct) (Zou et al.).

Sweet wormwood (*Artemisia annua*) has been employed in Chinese traditional medicine to treat malaria and consequently against the intra-cellular Plasmodium. What is not clear whether sweet wormwood *per se* has positive or negative effects on chickens. One study in the present topic addresses the ability of sweet wormwood leaf powder to ameliorate effects of a challenge with mixed *Eimeria* spp. in laying hens (Sharma et al.). This builds on the work of Jiao et al. (2018) As would be expected, egg production was decreased by a mixed *Eimeria* spp. Challenge (Sharma et al.). The effect was partially overcome by dietary supplementation with sweet wormwood leaf powder (Sharma et al.). While a challenge with mixed *Eimeria* spp. influenced the expression of a series of genes for intestinal proteins including mucin 2, tight junction proteins and transporter proteins and both small intestine villus height and crypt depth, there were no effects of supplementation with sweet wormwood leaf powder (Sharma et al.). Similarly, *Eimeria* challenge influenced anti-oxidative system but the effects were no ameliorated by supplementation with sweet wormwood leaf powder did not (Sharma et al.).

Ducks and geese are force-fed to produce those fatty livers needed for “Bloc foie gras” and “Pâté de foie gras”. There are increases in both liver weight and lipid percentage fat in *force fed mule ducks* (Caïrina moschata x *Anas platyrhynchos*) (Atallah et al.). There is hepatic steatosis with concentrations of lipids rising from about five percent to about 60% with increasing bouts of force feeding (Atallah et al.). There are other novel shifts in hepatic physiology such as with the anti-oxidant status*.* In both male and female mule ducks, hepatic activities (expressed per unit protein) of superoxide dismutase were increased while those of catalase were decreased with increasing bouts of force feeding (Atallah et al.). Moreover, there were decreases in glutathione (total, oxidized and reduced expressed per unit protein) but increases in hypoxia-inducible factors 1 and 2 (Atallah et al.). Atallah and colleagues (2024) also report in the effects of force feed on expression of a comprehensive series of gene. There were little changes in inflammatory related genes, interleukin 18 and tumor necrosis factor-α (TNF-α) or metabolic related genes (Atallah et al.). However, expression of fatty acid binding protein greater than eighty-fold with 21 bouts of force feeding (females: 121-fold, males 87-fold increase).

Fecal metabolites were quantified by H^1^ Nuclear Magnetic Resonance (NMR) spectroscopy in wild ducks (mallards) subjected to feed restriction (Murray and Machin). There were decreased fecal concentrations of O-phosphocholine, serine, taurine, caprate, ascorbate and increased fecal concentrations of 3-hydroxybutyrate, creatine, methyl amine, arabinitol, glutamic acid, glucuronate, trehalose, glucose and trimethylamine (Murray and Machin). This provides a tool to investigate nutritional status of mallards in the wild based on fecal samples.

## References

[B2] Jiao J. Y. Yang Y. Q. Liu M. J. Li J. G. Cui Y. Yin S. J. 2018. Artemisinin and Artemisia annua leaves alleviate *Eimeria tenella* infection by facilitating apoptosis of host cells and suppressing inflammatory response. Veterinary Parasitol. 254, 172–177. 10.1016/j.vetpar.2018.03.017 29657004

